# Identification and Characterization of a Liver Stage-Specific Promoter Region of the Malaria Parasite *Plasmodium*

**DOI:** 10.1371/journal.pone.0013653

**Published:** 2010-10-27

**Authors:** Susanne Helm, Christine Lehmann, Andreas Nagel, Rebecca R. Stanway, Sebastian Horstmann, Manuel Llinas, Volker T. Heussler

**Affiliations:** 1 Malaria Laboratory 1, Bernhard Nocht Institute for Tropical Medicine, Hamburg, Germany; 2 Carl Icahn Laboratory, Princeton University, Princeton, New Jersey, United States of America; New York University, United States of America

## Abstract

During the blood meal of a *Plasmodium*-infected mosquito, 10 to 100 parasites are inoculated into the skin and a proportion of these migrate via the bloodstream to the liver where they infect hepatocytes. The *Plasmodium* liver stage, despite its clinical silence, represents a highly promising target for antimalarial drug and vaccine approaches. Successfully invaded parasites undergo a massive proliferation in hepatocytes, producing thousands of merozoites that are transported into a blood vessel to infect red blood cells. To successfully develop from the liver stage into infective merozoites, a tight regulation of gene expression is needed. Although this is a very interesting aspect in the biology of *Plasmodium*, little is known about gene regulation in *Plasmodium* parasites in general and in the liver stage in particular. We have functionally analyzed a novel promoter region of the rodent parasite *Plasmodium berghei* that is exclusively active during the liver stage of the parasite. To prove stage-specific activity of the promoter, GFP and luciferase reporter assays have been successfully established, allowing both qualitative and accurate quantitative analysis. To further characterize the promoter region, the transcription start site was mapped by rapid amplification of cDNA ends (5′-RACE). Using promoter truncation experiments and site-directed mutagenesis within potential transcription factor binding sites, we suggest that the minimal promoter contains more than one binding site for the recently identified parasite-specific ApiAP2 transcription factors. The identification of a liver stage-specific promoter in *P. berghei* confirms that the parasite is able to tightly regulate gene expression during its life cycle. The identified promoter region might now be used to study the biology of the *Plasmodium* liver stage, which has thus far proven problematic on a molecular level. Stage-specific expression of dominant-negative mutant proteins and overexpression of proteins normally active in other life cycle stages will help to understand the function of the proteins investigated.

## Introduction

Malaria remains one of the main health burdens in developing countries, especially in sub-Saharan Africa. Much recent work on developing vaccines against the *Plasmodium* parasite, the causative agent of the disease, has concentrated on the liver stage of the parasite [1]. The liver stage is particularly attractive for vaccine development as it presents a chokepoint in parasite development where low numbers of parasites are present [2]. However, there is a great discrepancy between the *Plasmodium* liver stage being the main target of vaccine development against malaria and our knowledge of the biology of the parasite at this particular stage. To facilitate studies on the liver stage of development, we sought to generate a highly specific liver stage reporter.

Transcriptomic and proteomic analyses of the parasite at various life cycle stages suggest stage-specific regulation of gene expression but nothing is known about the regulation of transcription during the liver stage. Recently, the ApiAP2 transcription factor family has been suggested to regulate stage-specific gene expression in the human parasite *Plasmodium falciparum* [3], [4]. However, these *in vitro* investigations thus far have only been conducted on the blood stage of the parasite. Recently, ApiAP2 regulators for sporozoite and ookinete development *in P. berghei* have been characterized, showing for the first time that there are stage-specific transcription factors governing parasite development [5], [6]. However, no liver stage-specific promoters have thus far been described. There may indeed be a relatively small number of tight promoters specific for this stage, due to the similarities between the liver stage and other stages and particularly as the ultimate end of both liver and asexual blood stage development is the production of merozoites. The aim of this study was to identify in the rodent model parasite *P. berghei* a liver stage-specific promoter and to verify its stage-specific expression using GFP and luciferase reporter assays.

## Results

**Figure 1:**
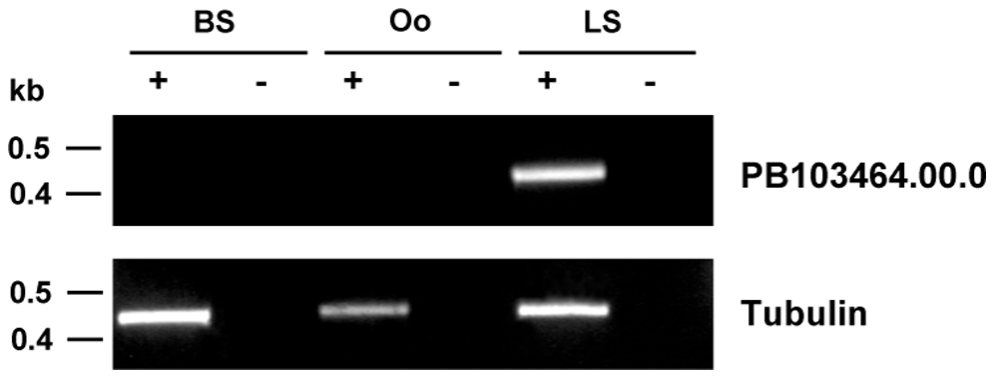
Liver stage-specific gene expression. (A) Results of RT-PCR analysis of PB103464.00.0 mRNA expression, comparing blood stage (BS), oocysts (Oo) and *in vitro* liver stage (LS) 48 hpi. Total RNA was extracted, and RT-PCR reactions were performed with (+) or without (−) reverse transcriptase (negative control). RT-PCR analysis of tubulin mRNA expression served as a control.

**Figure 2:**
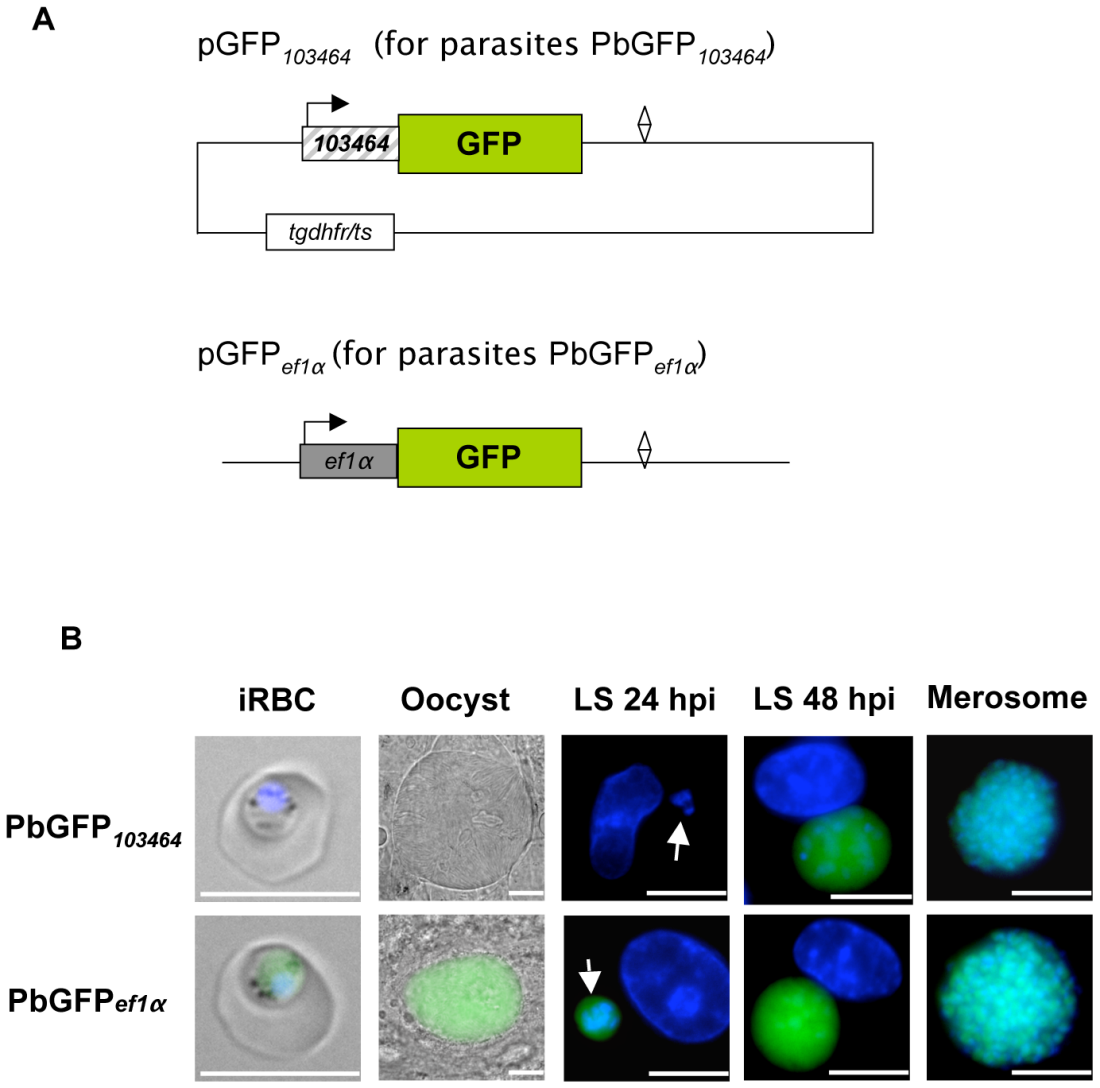
Promoter-dependent GFP expression. (A) The vector pGFP*_103464_* with GFP under the control of the promoter region *103464* and the vector pGFP*_ef1α_* with GFP under control of the *ef1α* promoter were generated and their transfection resulted in the parasite lines PbGFP*_103464_* and PbGFP*_ef1α_*. (B) Live imaging of PbGFP*_ef1α_* and PbGFP*_103464_* parasites at different life cycle stages. HepG2 cells were infected with transgenic *P. berghei* sporozoites and analyzed at different time points after infection (hpi, hours post-infection). GFP expresion was monitored by fluorescent microscopy. DNA was stained with Hoechst 33342. Arrows indicate young liver stage parasites. (iRBC: infected red blood cell; LS: liver stage) Scale bars: 10 µm.

In search of a truly liver stage-specific promoter, we analyzed mRNA expression of a number of genes predicted to be liver stage-specifically expressed in *Plasmodium yoelii* [7] and concentrated on PY05129. This gene has been identified in a recent DNA microarray study as expressed solely during the liver stage [8]. We chose to analyze the *P. berghei* ortholog of PY05129, PB103464.00.0 (PBANKA_100300 in GeneDB), which we find to also be exclusively expressed during the mid to late *P. berghei* liver stage (**Figure 1**). PB103464.00.0 and PY05129 have a close homology on an amino acid level and their upstream putative promoter regions also show a close similarity. The most recent release of PlasmoDB (version 7.0, September 2010) shows PBANKA_100300 to have syntenic homologs in all sequenced *Plasmodium* genomes, with the *P. falciparum* homolog being PFD0260c. Between the start codon of the PB103464.00.0 gene and the next gene upstream, a region of 989bp is present. (**Supplementary Figure S1**). We concluded that the minimal promoter of PB103464.00.0 is located within this region and cloned the −989/+1 gDNA fragment into a *P. berghei* transfection plasmid upstream of *gfp* (**Figure 2A**, upper panel) to confirm its stage-specific activity. The generated plasmid pGFP*_103464_* was transfected into *P. berghei* and a parasite strain, PbGFP*_103464_*, was established. A parasite line in which GFP is constitutively expressed under the control of the *pbeef1αa* promoter (abbreviated in plasmid and parasite names as *ef1α*) was chosen as a control for promoter activity (**Figure 2A**, lower panel). Live imaging of blood stages, oocysts and of *in vitro* liver stages was performed. GFP expression driven by the PB103464.00.0 5′ upstream region was restricted to the liver stage (**Figure 2B**, upper panel), confirming that the chosen 5′ upstream region indeed contains a liver stage-specific promoter. As expected, GFP expression under the control of the constitutive *pbeef1αa* promoter was detectable in all stages analyzed (**Figure 2B**, lower panel). During parasite development in hepatocytes, GFP expression under the PB103464.00.0 promoter was strongly upregulated at 48 hpi and continued until merosome formation, the end-point of liver stage development.

**Figure 3:**
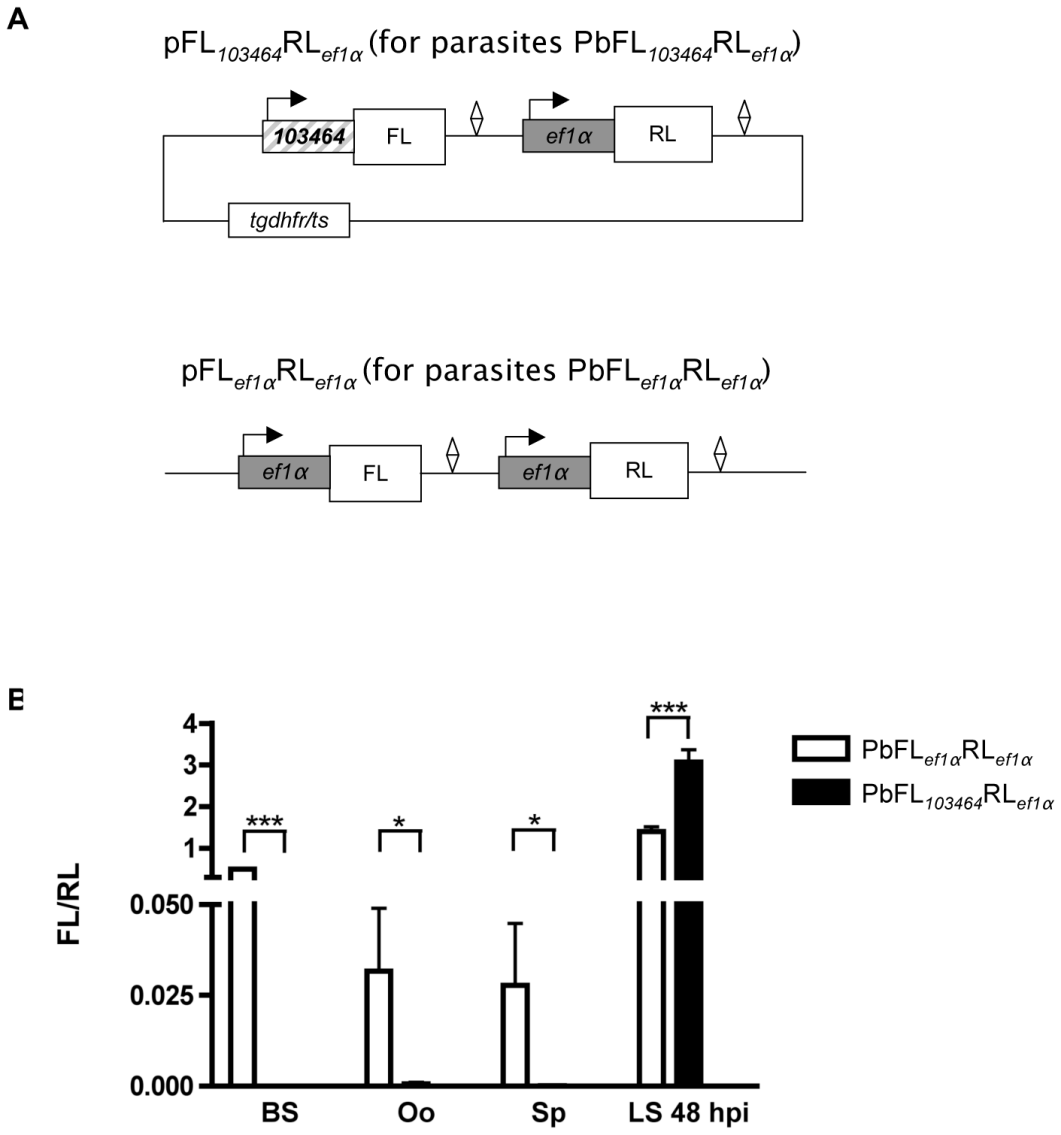
Generation of plasmids and transgenic *P. berghei* parasites for use in dual-luciferase assays. (A) The vector pFL*_103464_*RL*_ef1α_* with FL under the control of the promoter region *103464* and RL under the control of the *ef1α* promoter was generated and its transfection resulted in the parasite line PbFL*_103464_*RL*_ef1α_*. The plasmid pFL*_ef1α_*RL*_ef1α_* with FL and RL under the control of the *ef1α* promoter was used to obtain control parasites PbFL*_ef1α_*RL*_ef1α_*. Diamonds represent 3′UTRs, which in all cases were from the *pbdhfr/ts* gene. In the upper plasmid, the selection marker (*tgdhfr/ts*) is displayed but for simplicity in all other plasmid diagrams only those genes and features directly related to the experiments described are displayed. (B) Comparison of the luciferase activity (FL expression relative to RL expression) of transgenic parasites during the blood stage (BS), in oocysts (Oo), in salivary gland sporozoites (Sp) and *in vitro* in the liver stage (LS), 48 hours post-infection (hpi) of hepatoma cells. Standard deviation values (shown as error bars) were determined from three different measurements. Statistical analysis was performed using two-tailed unpaired t-tests (*P<0.05; ***P<0.001).

Although the GFP expression findings supported our mRNA transcription studies, the analysis of GFP expression has its limitations as it does not allow an accurate quantitative analysis of promoter activity. We therefore decided to clone the promoter region in front of the firefly luciferase (FL) gene [9] and to normalize the measurement of firefly luciferase activity by including in the same plasmid a *Renilla* luciferase (RL) gene [10] under the control of a constitutive promoter (**Figure 3A**). This plasmid was named pFL*_103464_*RL*_ef1a_*. As a control, a further plasmid was constructed in which both luciferase genes were under the control of the constitutive promoter (pFL*_ef1a_*RL*_ef1a_*) (**Figure 3A**). Both plasmids were transfected separately into *P. berghei* and the parasite strains PbFL*_103464_*RL*_ef1a_* and PbFL*_ef1a_*RL*_ef1a_*, were established. The plasmids used allow integration into the *P. berghei d*/*c ssu rRNA* locus, but are also able to persist in the parasite population as episomes. As both luciferases are encoded on each plasmid and therefore the genes are always present in equal numbers, it was not necessary to analyze genetically whether the parasite populations contained integrated or episomal gene copies or (as is most likely) a mixture of both. Transfection of a single plasmid carrying both luciferase genes has great advantages over traditional co-transfection of two plasmids each containing a single luciferase gene, which results in a mixture of single- and double-transfectants, meaning that cloning must be performed to produce a population where each plasmid is integrated only once. In *P. berghei*, such limiting dilution cloning requires the use of numerous mice. As transfection with the single plasmids described above results in parasites that contain equal copy numbers of each gene, this strategy greatly simplifies later analysis and also potentially saves large numbers of mice.

Using the above parasite strains, we determined the relative expression level of FL compared to RL in the blood stage, in oocyst and salivary gland sporozoites and in the liver stage (in infected hepatoma cells) at 48 hpi using a dual-luciferase assay (**Figure 3B**). FL expression of the parasites PbFL*_103464_*RL*_ef1α_* was absent during the blood and mosquito stage and was seen to only be high during the liver stage, confirming the GFP expression results and also the transcription profile of the PB103464.00.0 gene determined by RT-PCR (**Figures 1** **,** **2**). Most importantly, the luciferase assays clearly show that the PB103464.00.0 promoter region is very tightly silenced during blood and insect stages. During the liver stage *in vitro*, the PB103464.00.0 promoter region is significantly more active at 48 hpi than the constitutive *pbeef1αa* promoter (*P*≤0.001 by two-tailed unpaired t-test). The PbFL*_ef1α_*RL*_ef1α_* parasites, in which both luciferase genes are independently expressed under the *pbeef1αa* promoter showed constitutive expression of both luciferase genes. Unexpectedly, however, the ratios of FL/RL expression varied at different life cycle stages. The reason for this variation is not clear but since both luciferase genes are transcribed under the control of the same constitutive promoter, it is likely that differing protein stability of the two luciferases at different life cycle stages is responsible.

**Figure 4:**
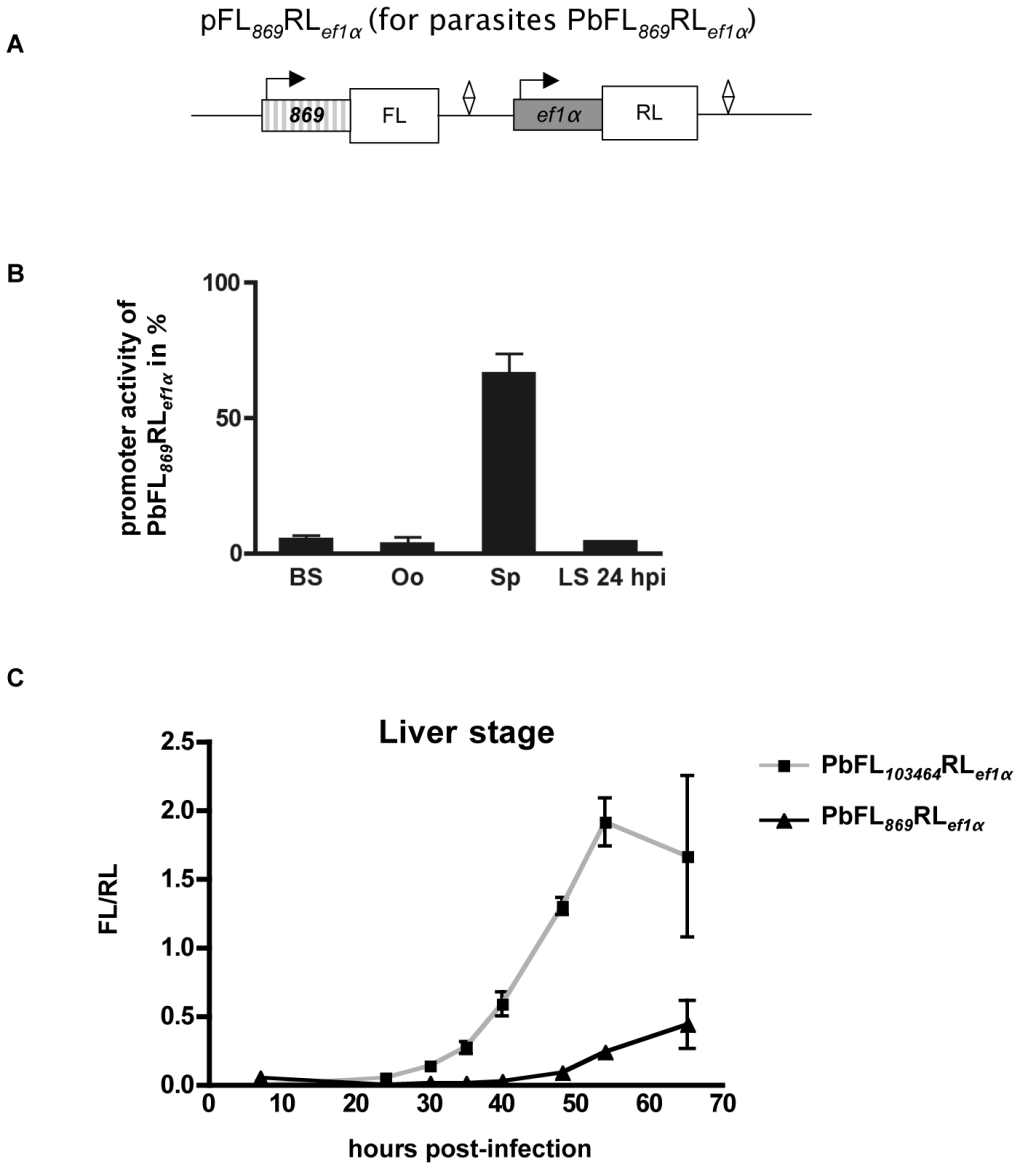
Comparison of the activities of promoter regions of the genes PB000869.01.0 and PB103464.00.0 during different life cycle stages. (A) Plasmid map of pFL*_869_*RL*_ef1α_*, used to generate the parasites PbFL*_869_*RL*_ef1α_*, which express *Renilla* (RL) and firefly (FL) luciferases under the control of the *pbeef1αa* and the PB000869.01.0 promoter region, respectively. The diamond represents the 3′UTR of the *pbdhfr/ts* gene. (B) PB000869.01.0 promoter activity in respect to the constitutive promoter *pbeef1αa*, which was set to 100%. PbFL*_869_*RL*_ef1α_* and PbFL*_ef1α_*RL*_ef1α_* parasites were harvested at different life cycle stages (BS; blood stage, Oo; oocysts, Sp; salivary gland sporozoites, LS 24 hpi; liver stage 24 hours post-infection *in vitro*), the ratio of FL to RL expression was determined and the percentage of luciferase activity of PbFL*_869_*RL*_ef1α_* parasites was calculated relative to that of PbFL*_ef1α_*RL*_ef1α_* parasites. (C) Comparison of luciferase activity in the parasites PbFL*_103464_*RL*_ef1α_* and PbFL*_869_*RL*_ef1α_* during the liver stage, as determined by the ratio of FL to RL expression. Standard deviation values (shown as error bars) were determined from three different measurements.

As an additional control, we decided to compare the liver stage-specific promoter to a promoter that is mainly active in another parasite stage. To this end, we first performed database searches and RT-PCR assays and identified PB000869.01.0 (PBANKA_040200, abbreviated in plasmid and parasite names as 869), as being expressed at a low level in the liver relative to other stages (data not shown). We cloned the 5′ upstream region of this gene in front of the FL resulting in the plasmid pFL*_869_*RL*_ef1α _*(**Figure 4A**). Transfection of this plasmid into *P. berghei* resulted in the generation of the parasite strain PbFL*_869_*RL*_ef1α_*. The PbFL*_869_*RL*_ef1α_* parasites showed a high firefly luciferase expression in sporozoites but nearly no FL activity in the blood and oocyst stage as well as *in vitro* in early liver stages (**Figure 4B**). FL/RL expression in these parasites was compared to that of PbFL*_103464_*RL*_ef1α_* parasites (**Figure 4C**). Whereas PbFL*_103464_*RL*_ef1α_* parasites exhibited an increasing FL activity in the liver stage from 24 hpi onwards, FL activity for PbFL*_869_*RL*_ef1α_* parasites was only found at 48 hpi and later stages, which roughly correlates with the onset of merozoite formation. At very late stages (detached cells/merosomes), FL activity of the PbFL*_103464_*RL*_ef1α_* parasites appeared to again decrease, probably reflecting the development of blood cell-infective merozoites.

**Figure 5:**
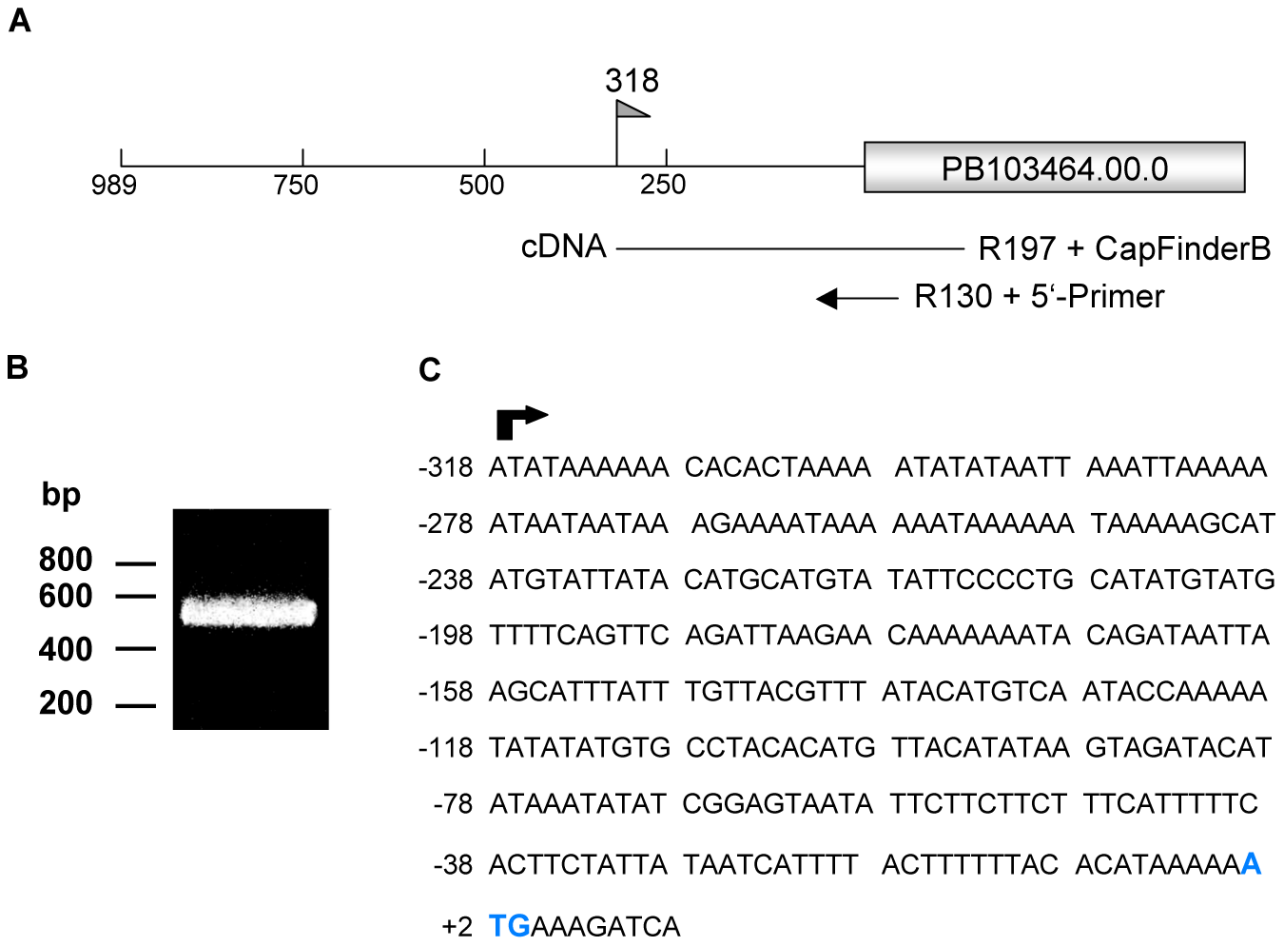
Identification of the transcription start site (TSS) of the promoter region of PB103464.00.0. (A) Schematic representation of the promoter region. A flag at position −318 shows the TSS. The primers used for the 5′-RACE are indicated. (B) The RACE product, obtained from the second PCR amplification, was analyzed by gel electrophoresis. (C) The 5′ UTR of PB103464.00.0 with the start codon, shown in bold. The arrow indicates the TSS of the promoter region.

**Figure 6:**
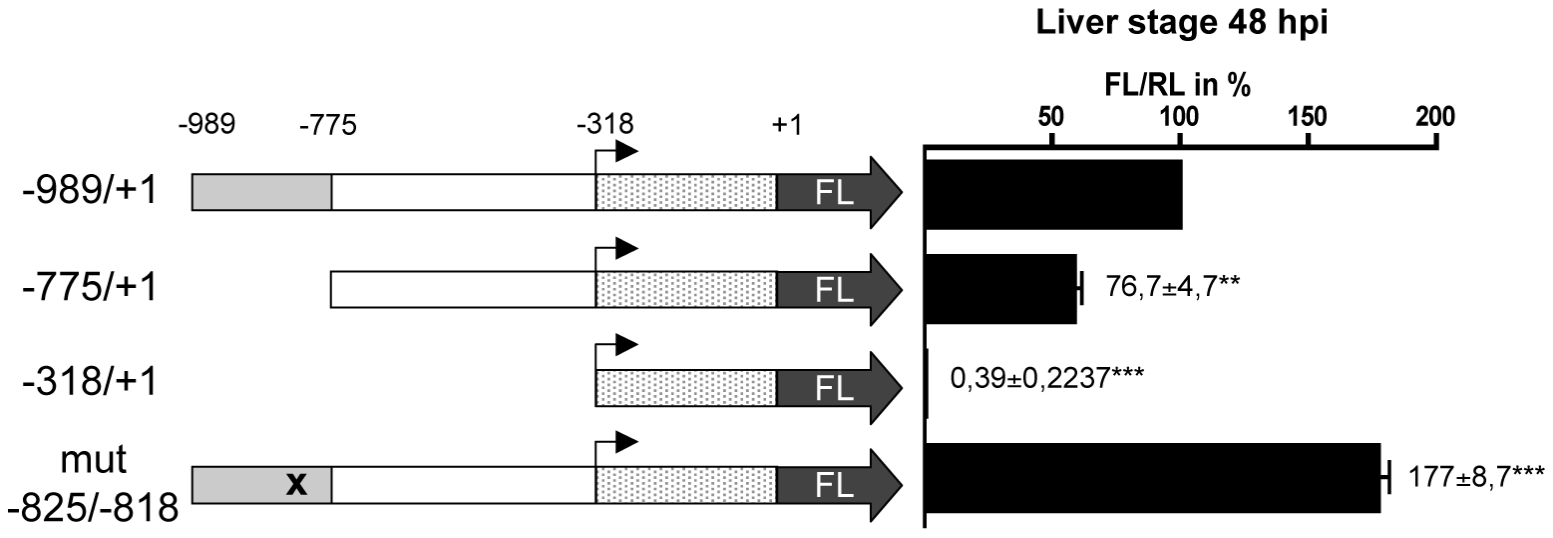
Promoter deletion affects expression of FL. Parasites transfected with the plasmid pFL*_103464_*RL*_ef1α_* carrying the complete promoter region (−989/+1), promoter deletions (PbFL*_103464(−775)_*RL*_ef1a_* (−775/+1) and PbFL*_103464(−318)_*RL*_ef1a_* (−318/+1)) or a promoter mutation (PbFL_103464(*825)_RL*_ef1a_*mut-825/−818)**were passed through mosquitoes and used for HepG2 cell infections. 48 hpi cells were harvested and dual-luciferase assays of the cell extracts were performed. The FL/RL ratio obtained from extracts of parasites transfected with the plasmid pFL*_103464_*RL*_ef1α_* was set to 100% and the FL/RL ratio obtained from the parasite strains transfected the other constructs was calculated.

Having confirmed a strict liver stage-specific activity of the promoter region of the gene PB103464.00.0 (−989/+1) we sought to determine possible transcription factor binding sites in this region. Since *Plasmodium* mRNAs often contain long 5′ untranslated regions (UTRs), which are unlikely to provide potential binding sites for transcription factors we first identified the transcription start site (TSS) for PB103464.00.0. Using 5′ RACE technology to amplify the 5′ untranslated region (**Figure 5A**) the RACE products were sequenced, revealing a TSS at position −318 (**Figure 5B, C**) restricting the DNA region interacting with transcription factors to between position −989 to −318. By bioinformatic analysis, we searched this region for sequence motifs known in *P. falciparum* to allow binding of different ApiAP2 transcription factors [4] and identified four potential binding sites for PB000252.02.0 (PBANKA_090960 in GeneDB, homolog of PF11_0404), although additional non-ApiAP2 binding sites might also exist (**Supplementary Figure S2**). To analyze the effect of the individual binding sites it is important to determine the basal transcription in the absence of these sites. To this end the −318/+1 region was cloned in front of the FL gene in the dual luciferase plasmid (pFL*_103464(-318)_*RL*_ef1α_*). Transfection of this plasmid into *P. berghei* resulted in the generation of the parasite strain PbFL*_103464(-318)_*RL*_ef1α_*. This parasite strain did not show a significant FL activity in liver stage parasites (**Figure 6**) or any other parasite stage investigated, confirming that the actual promoter region must be localized between position −989 and −318. We next generated the parasite strain PbFL*_103464(−775)_*RL*_ef1α_* to analyze whether the promoter region is positioned between −775 and +1. In this region two of the four predicted PB000252.02.0 ApiAP2 binding sites were found. Two additional sites were found between the excluded region −989 and −775 (**Supplementary Figure S2**). We detected a significant but not complete reduction in FL activity in the PbFL*_103464(−775)_*RL*_ef1α_* parasites compared to the PbFL*_103464_*RL*_ef1α_* parasites, suggesting that both the excluded sequence −989/−775 and the −775/+1 region contain functional portions of the promoter. Deletion experiments do not allow the exact identification of transcription factor binding sites and therefore we mutated one predicted ApiAP2 binding site (at position −825/−818), with the sequence TAGAACA [4]. To our surprise, the mutation resulted in an increased luciferase activity, suggesting that the transcription factor binding to this particular ApiAP2 site acts as a repressor (**Figure 6**). Interestingly, the mutation did not result in increased luciferase levels in other parasite stages and so the liver stage-specific transcription profile was maintained.

Our data have identified a promoter region derived from PB103464.00.0 that is able to direct exclusively liver-stage expression of genes of interest. In future experiments we will investigate the role of the predicted transcription factor binding sites in more detail as well as seeking to identify additional sites by bioinformatic and experimental approaches.

## Discussion

Several liver stage-specifically expressed genes have been described in the literature [11], [12], [13], [14] but previous studies have not analyzed the promoters of these genes. We describe here for the first time a promoter region that is only active during the liver stage. *In vitro* it is only possible to achieve relatively low rates of hepatoma cell infection by *Plasmodium* sporozoites, which makes biochemical analysis of the liver stage very difficult. Luciferase reporter assays have proved to be extremely useful for the detection of minute numbers of parasites in the blood of infected mice [15] and therefore we decided to employ a dual-luciferase assay to quantitatively analyze promoter activity *in vitro*. Using this very sensitive technique, it is possible to analyze promoter activity of any given gene in all life cycle stages.

The results of this study verified that the −989/−318 promoter region of PB103464.00.0 is sufficient for gene expression to be restricted to the liver stage. This is perhaps surprising since *Plasmodium* parasites have been seen to use other *cis*-regulatory elements around the TSS, in introns and in the 3′ region of genes to control mRNA expression [16], [17], [18], [19]. Epigenetic alterations, which might differ substantially in the transfected versus the endogenous promoter region, due to their differing positions within the genome, seem not to play an important role in the case of the PB103464.00.0 promoter region.

We further showed that the deletion of the −989/−775 region caused a significant reduction in but not complete abolishment of luciferase expression, suggesting that the minimal promoter includes distantly situated motifs. Interestingly, the deletion did not result in an altered expression profile in other parasite stages indicating that the remaining −775/+1 region is sufficient to guarantee liver stage-specific expression, albeit at a reduced level.

Bioinformatic analysis of the entire −989/−318 region revealed the existence of four putative binding sites that could be recognized by the ApiAP2 protein PB000252.02.0, based on homology with the *P. falciparum* protein PF11_0404. Two were found in the −989/−775 region and the two others in the −775/−318 region. It has been suggested that ApiAP2 transcription factors are involved in stage-specific gene regulation since several *P. falciparum* ApiAP2 proteins show no detectable gene expression during the blood stage [4]. It is tempting to speculate therefore, that these ApiAP2 proteins would play important roles during either the mosquito or liver stages. Two reports have already documented that the elimination of a single ApiAP2 gene is sufficient to prevent the development of either ookinetes or sporozoites [5], [6]. Interestingly, homologs of these genes in *P. falciparum* are expressed additionally in the asexual stages, implying that they have roles across diverse stages. Of course, it is possible that other transcription factors might also be involved in regulating expression via the −989/+1 region, including the canonical TATA-box-binding protein that has been identified in the *P. falciparum* genome [19], [20]. The finding that a mutation of one predicted ApiAP2 binding site in our promoter of interest lead to an increase in luciferase activity suggests that ApiAP2 proteins could have not only an activator but also a repressor function in the control of PB103464.00.0 expression.

A systematic dissection of the PB103464.00.0 promoter region is a major undertaking since it includes the generation of numerous parasite strains, which must all be generated by transfection of blood stage schizonts, requiring many mice. Additionally, the parasites need passage through mosquitoes before they can finally be used to infect hepatic cell lines. Sequential deletion of promoter regions will provide useful information about the possible transcription factor binding sites. However, since our results suggest that several regions are likely involved in regulating expression and because deletions affecting the distance of putative elements could disturb the functionality of putative control elements, other techniques such as linker scanning approaches may also have to be applied [21]. Bioinformatic approaches to search for motifs common to exclusively liver stage promoters will allow for focused mutation-based strategies, limiting the number of parasite strains to be generated and hence the usage of mice. A prerequisite for this approach is the identification of other liver stage-specific promoters and their comparison with the PB103464.00.0 promoter region. In a first attempt, we compared this promoter with the 5′ upstream region of the liver stage specific protein LISP1 [22] and found that the LISP1 promoter, like that of PB103464.00.0, contained putative binding sites for the ApiAP2 protein PB000252.02.0 (the homolog of PF11_0404). However, no other homologous regions were found by simple comparison of the promoters and it might be necessary to employ other methods to identify additional transcription factor binding sites.

Proteins expressed solely at the liver stage are themselves interesting for study and might be useful candidates for subunit vaccine approaches. These proteins are likely to reveal novel liver stage-specific pathways or metabolic processes that could be targeted to eliminate or attenuate the parasite during this stage. For example, it has recently been demonstrated that fatty acid metabolism is essential only for liver stage parasites but not for other stages [23]. Given the current focus on the possibility of vaccines targeted against liver stage parasite development, using either radiation or genetically attenuated parasites, such studies are likely to provide new leads for vaccine development.

To better understand the biology of the *Plasmodium* liver stage, it is essential to analyze the functions of proteins expressed at this stage. To this end reverse genetics stands out as an excellent tool, although its utility is frequently complicated in *Plasmodium*. Transfection must be performed during the blood stage and any genes with an essential function here cannot be targeted by traditional knockout approaches [24]. For study in the liver stage, the protein of interest must also be dispensable during development within the mosquito. Recently a very elegant system has been developed that allows the control of the expression levels of a protein of interest by its fusion with a destabilization domain. This system has been used successfully in *Plasmodium falciparum* blood stage parasites [25]. However, the system relies on the constant application of the ligand Shld1, which binds to the fused destabilization domain, until protein degradation is required and as this is unlikely to be possible during mosquito stages, the method cannot yet be used for studying the consequences of protein depletion during either the mosquito or liver stages. Conditional or inducible knockouts are useful methods to study essential genes [24], [26] but these systems rely either on the stage-specific expression of a recombinase or other regulatory proteins. The liver stage-specific promoter described in this study might be useful for the expression of such proteins. A liver stage-specific promoter could also be used for the expression of dominant-negative mutant proteins to study protein function specifically in the liver stage or for the expression of proteins that cause adverse effects on the parasite when expressed constitutively. In summary, the identification and the use of a liver stage-specific promoter of *P. berghei* will have a great impact in the analysis of the parasite liver stage, an area fundamentally lacking in understanding.

## Materials and Methods

### Experimental animals and parasites

Mice used in experiments were of the NMRI strain, between 6 and 10 weeks of age and were bred in-house at the Bernhard Nocht Institute for Tropical Medicine (Hamburg, Germany) or supplied by Charles River Laboratories. All animal work was conducted in compliance with regulations created and approved by the ethical committee of Hamburg state authorities (Nr. FI 28/06).

### RT-PCR

Total RNA was isolated from 0.05% saponin-treated (Sigma) blood stage parasites, 20 infected mosquito midguts day 14 after infection and infected HepG2 cells 48 hours after infection using the NucleoSpin® RNA Extract Kit II (Macherey-Nagel). First strand cDNA was synthesized using random primers (Invitrogen) and Superscript™II reverse transcriptase (Invitrogen) according to the manufacturer's instructions. Target cDNAs were amplified using GoTaq® DNA polymerase (Promega) and the following primer sets: 5′-AACAGCAATATATCGTCACCAAG-3′ and 5′-GCACACGGAAATCATTTTGTT-3′. As in internal control, *P. berghei* tubulin cDNA was amplified using the primer 5′-TGGAGCAGGAAATAACTGGG-3′ and 5′- ACCTGACATAGCGGCTGAAA-3′.

### Mapping of the transcription start site (5′-RACE)

The transcription start site (TSS) of the promoter region *103464* was determined by 5′ rapid amplification of cDNA ends (5′-RACE) based on the CapFinder method by Schramm et al., 2000 [27]. 1 µg RNA of *P. berghei* infected HepG2 cells isolated 48 hpi (NucleoSpin® RNA II Kit, Macherey-Nagel) was reversed transcribed by Superscript™II (Invitrogen) using a gene-specific primer R197 5′-ACATCCGTATTTTTCCTATTGACA-3′ and the CapFinderB [27]. cDNA was then amplified by PCR using a gene-specific nested reverse oligonucleotide R130 5′-TGCTGTTGTATTTTTGTTTTTCATC-3′ and the 5′-Primer [27]. The amplification products were cloned into the pGEM®-T easy vector (Promega), DNA was extracted from 10 individual transformants and sequenced.

### Cell culture and *in vitro* infection of HepG2 cells

Human hepatoma cells (HepG2) were obtained from the European cell culture collection and were maintained in complete MEM (cMEM) with Earle's Salts Medium supplemented with 10% heat-inactivated FCS (foetal calf serum), 1% L-Glutamine, 1% penicillin/streptomycin (all purchased from PAA Laboratories, Austria). Cells were kept at 37°C in a 5% CO_2_ cell incubator and split every 3–4 days by trypsinization.

1×10^5^ HepG2 cells were seeded into glass bottom dishes (WillCo Wells BV, Netherlands) for live cell imaging or 5×10^4^ HepG2 cells into each well of a 24 well plate. Sporozoites were prepared from dissected salivary glands of *P. berghei-*infected *Anopheles stephensi* mosquitoes and incubated in media with HepG2 cells for 1–2 hours. After washing, the cells were incubated with cMEM, as described above, containing Amphotericin B at 2.5 µg/ml (PAA Laboratories, Austria) at 37°C and 5% CO_2_ for indicated times.

### Live cell imaging and live staining

Live imaging was performed with a Zeiss Axiovert 200 inverted microscope and images taken and processed with the Improvision software Openlab 5.0.1. Cell and parasite DNA staining was carried out by incubation with 1 µg/ml Hoechst 33342 (Sigma) for 20 min at 37°C and 5% CO_2_.

For live imaging of *P. berghei* blood stages, tail blood was taken from an infected mouse, mixed with room temperature cMEM (see above) containing 1 µg/ml Hoechst 33342 (Sigma) and microscopically analyzed. Midguts of infected *A. stephensi* mosquitoes were dissected for live imaging and microscopically investigated.

### Dual-luciferase assays

To determine the blood stage luciferase expression in the transgenic *P. berghei* lines, 10 µl of tail blood of an infected mouse was lysed for 15 min at 30°C with 100 µl 1x Passive Lysis Buffer (PLB) obtained from the Dual-Luciferase® Reporter (DLR™) Assay System (Promega). For measuring luminescence in the mosquito stage of the parasite, 5 midguts (day 14 after infection) or salivary glands of three infected *A. stephensi* mosquitoes were removed and lysed with 100 µl 1x PLB as above. For analyzing the liver stages of the transgenic *P. berghei* lines, HepG2 cells were infected with sporozoites (see above). At different time points after infection the culture medium was removed and the cells washed once with PBS, before lysis in 100 µl 1x PLB for 15 min at 30°C. Samples were processed according to the protocol of the kit. Briefly, after centrifuging the samples for 30 s at 12,000 g, 20 µl of the supernatant was added to 100 µl of Luciferase Assay Reagent II. Luminescence was measured in a single tube Junior LB 9509 luminometer (Berthold Technologies). After a 10 second measurement period 100 µl 1x Stop & Glo® Reagent was added to the tube to stop the firefly and activate the *Renilla* luminescence. The output of the luciferase assays was in relative light units (RLU). All assays were performed in triplicate. Either uninfected mosquitoes or HepG2 cells were used as negative controls, as appropriate. Samples can be collected and stored at −20°C to perform one combined dual luciferase assay.

For calculation of the promoter activity, the mean RLU value of control samples was subtracted from the mean RLU value of experimental samples. The ratio of the firefly and the *Renilla* luciferase RLUs indicated the activity of the promoter of interest. Promoter activity curves were generated and statistical analysis of the data performed using MS Office Excel and the GraphPad Prism software (GraphPad Prism software Inc., US). Significance levels were calculated with two-tailed unpaired t-tests.

### Generation of transgenic *P. berghei* parasites

For analyzing the promoter region of PB103464.00.0, the forward oligonucleotide 5′-CGGATATCGTTGCATTATCGTCAAAAGTG-3′ and the reverse oligonucleotide 5′-CGGGATCCTTTTTATGTGTAAAAAAGTAAAATGATT-3′ were used to amplify a fragment of 989 bp by PCR using Phusion® Taq High-Fidelity DNA polymerase (Finnzyme) from *P. berghei* gDNA (underlined nucleotides represent *EcoR*V and *BamH*I restrictions sites, respectively). The promoter region of PB103464.00.0 was used to replace the *pbeef1αa* promoter in *EcoR*V-*BamH*I-digested pL0017 vector (obtained through the MR4 (MRA-786), deposited by C. Janse) to create the plasmid pGFP*_103464_*.

For the dual-luciferase assay, plasmids with the firefly (FL) and the *Renilla* (RL) luciferase were generated. The firefly luciferase gene was amplified by PCR from pBI-GL (Clontech) and was used to replace the GFP in the pL0017 vector to generate pFL*_ef1α_*. The same procedure was performed to generate the plasmid pRL*_ef1α_*, amplifying the *Renilla* luciferase (RL) gene by PCR from phRL-CMV (Promega). The *pbeef1αa* promoter of the pFL*_ef1α_* plasmid was replaced with the promoter region of PB103464.00.0 to create plasmid pFL*_103464._* For the generation of the double-luciferase plasmid, the entire cassette for RL expression under the control of the *pbeef1αa* promoter (including the 3′ UTR of *Pbdhfr*) was amplified by PCR and ligated into pFL*_103464_* and pFL*_ef1α_* plasmids to generate pFL_103464_RL*_ef1α_* and pFL*_ef1α_*RL*_ef1α_*, respectively.

The plasmid pFL*_869_*RL*_ef1α_* was generated similarly having first amplified fragment 869, a 1 kb fragment upstream of PB000869.01.0, by PCR using the primers 5′-GGCCGCGGCCGCGTCTAAAGCATACAATAACTCTTAC-3′ and 5′-CTAGCCTAGGTTTGTATATTTCTGAGATTCCAAAAAAA-3′.

For further analysis of the 103464 promoter region, the 5′-truncation fragments of the promoter were obtained by PCR using the reverse primer 5′-CGGGATCCTTTTTATGTGTAAAAAAGTAAAATGATT-3′ and either the forward primer 5′-GCGCGGCCGCAAAATAAAACGAATAACGATGTGA-3′ (truncation -775/+1) or 5′-GCGCGGCCGCATATAAAAAACACACTAAAAATATATAATTAAAT-3′ (truncation −318/+1) (underlined nucleotides represent *Not*I restriction site). Mutagenesis PCR was performed to mutate the putative ApiAP2 binding site at −825/−818 of the 103464 promoter from TAGAACAA to TTATTATT. The truncated and mutated promoter fragments were used the replace the original promoter region of PB103464.00.0 in the plasmid pFL*_103464_*RL*_ef1α_* and transfection resulted in the parasites PbFL*_103464(−775)_*RL*_ef1α_,* PbFL*_103464(−318)_*RL*_ef1α_* and PbFL_103464(*825)_RL*_ef1α._*

*P. berghei* schizont stages (ANKA strain) were transfected with *Apa*I-*Sac*II-linearized plasmid DNA (5–10 ng). Transgenic parasites were selected by pyrimethamine treatment [28].

## Supporting Information

Figure S1 Detailed cloning strategy for testing activity of the PB103464.00.0. promoter region. The entire region between gene PB103463.00.0. and PB103464.00.0. was cloned in the plasmid pL0017 in front of the gfp cDNA.
(0.08 MB TIF)

Figure S2 DNA sequence of the PB103464.00.0. promoter region. The transcription start site at position -318 and potential ApiAP2 binding sites are labeled in colours. Since no data are available on *P. berghei* ApiAP2 transcription factors, the *P. falciparum* ApiAP2s, which would bind the indicated sequences are depicted.
(1.30 MB TIF)
