# Supplementary figures and images for: Identification and Characterization of a Liver Stage-Specific Promoter Region of the Malaria Parasite Plasmodium
Source: PLoS One. 2010 Oct 27;5(10):e13653. doi: 10.1371/journal.pone.0013653 (PMC2965107; doi:10.1371/journal.pone.0013653)

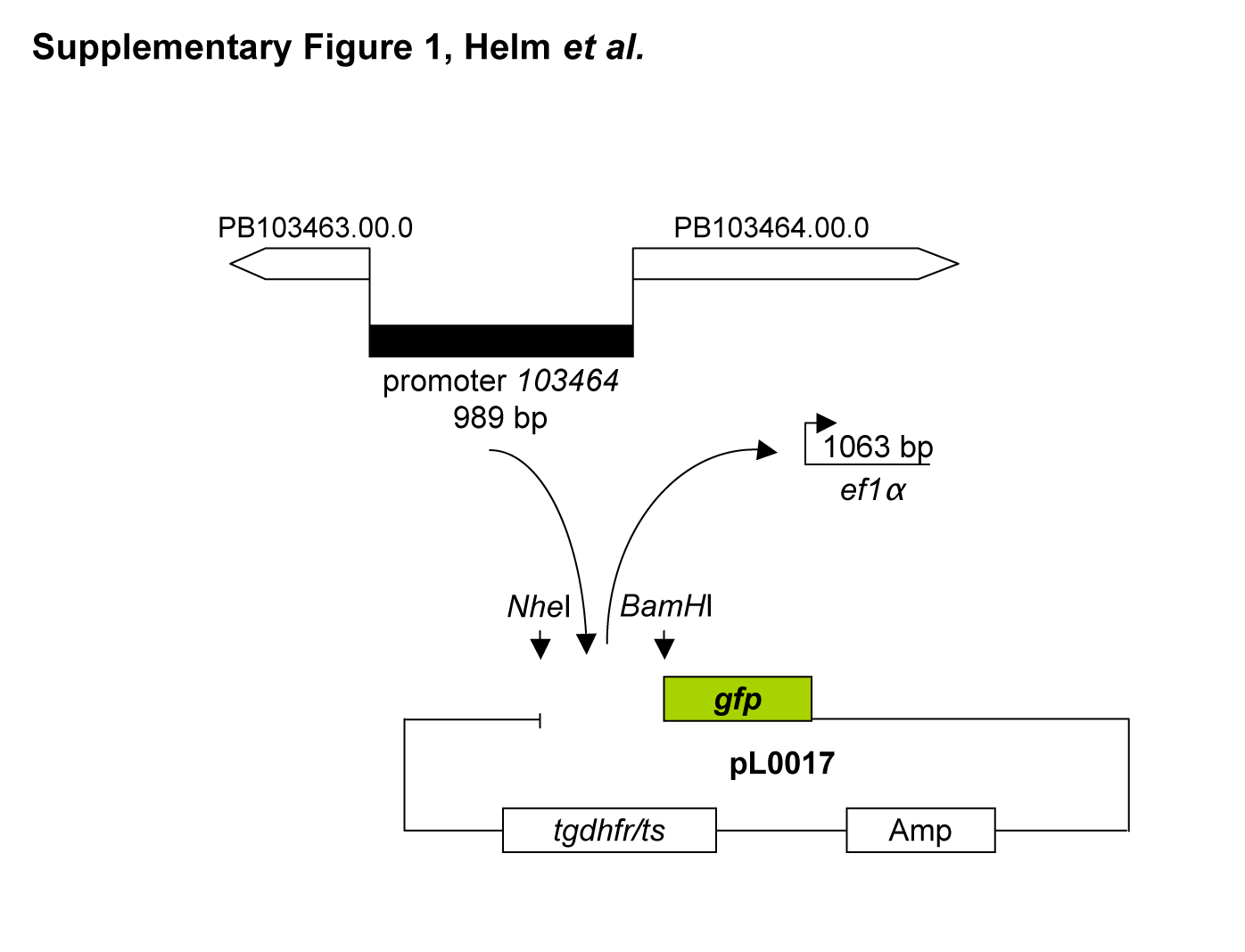

Supplement: Figure S1 — Detailed cloning strategy for testing activity of the PB103464.00.0. promoter region. The entire region between gene PB103463.00.0. and PB103464.00.0. was cloned in the plasmid pL0017 in front of the gfp cDNA. (0.08 MB TIF) [file pone.0013653.s001.tif]

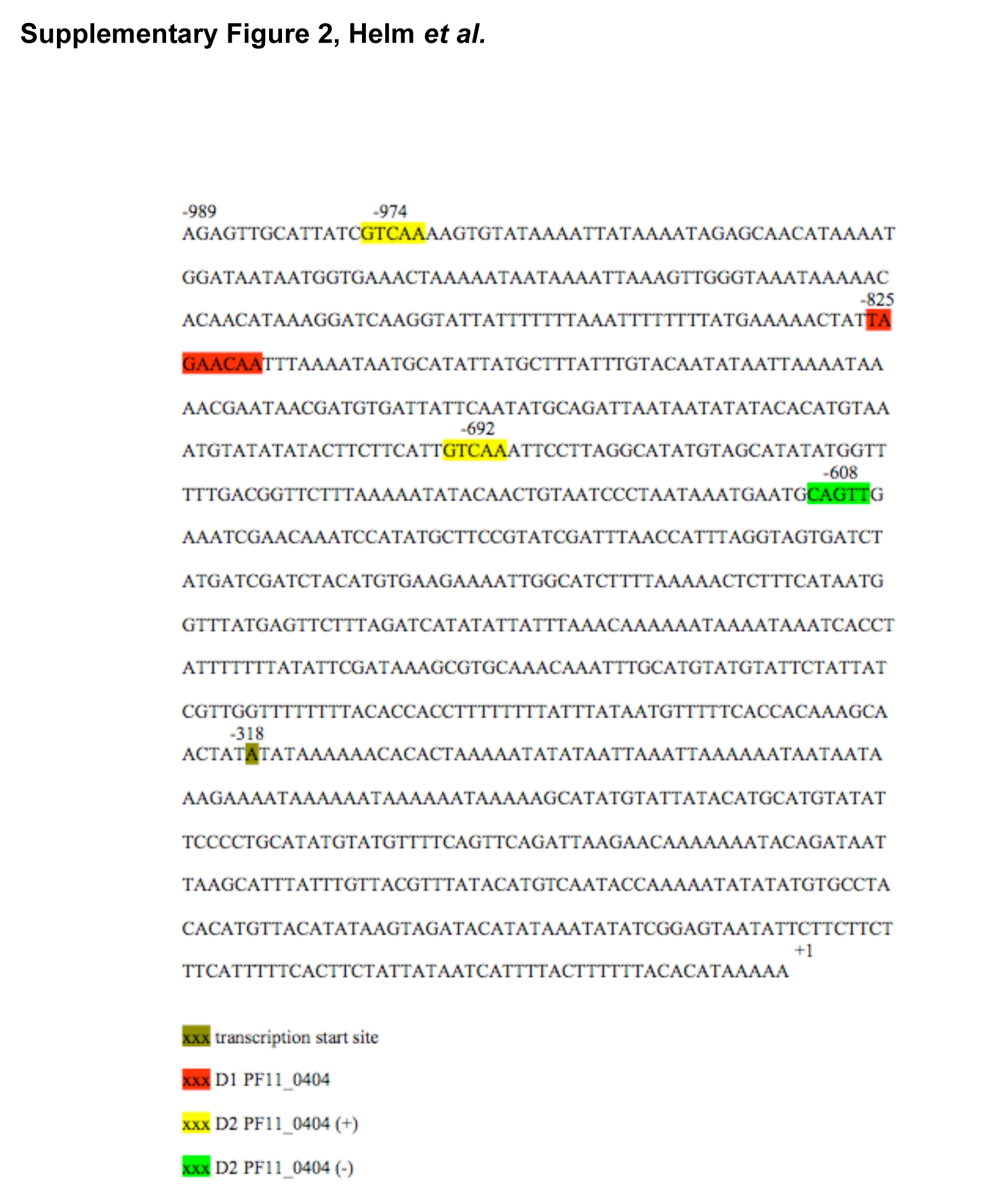

Supplement: Figure S2 — DNA sequence of the PB103464.00.0. promoter region. The transcription start site at position -318 and potential ApiAP2 binding sites are labeled in colours. Since no data are available on P. berghei ApiAP2 transcription factors, the P. falciparum ApiAP2s, which would bind the indicated sequences are depicted. (1.30 MB TIF) [file pone.0013653.s002.tif]
